# Supplementary material for: Two decades of neuroscience publication trends in Africa
Source: Nat Commun. 2021 Jun 8;12:3429. doi: 10.1038/s41467-021-23784-8 (PMC8187719; doi:10.1038/s41467-021-23784-8)
Supplement: Supplementary file 1 — Supplementary information. [file 41467_2021_23784_MOESM1_ESM.pdf]

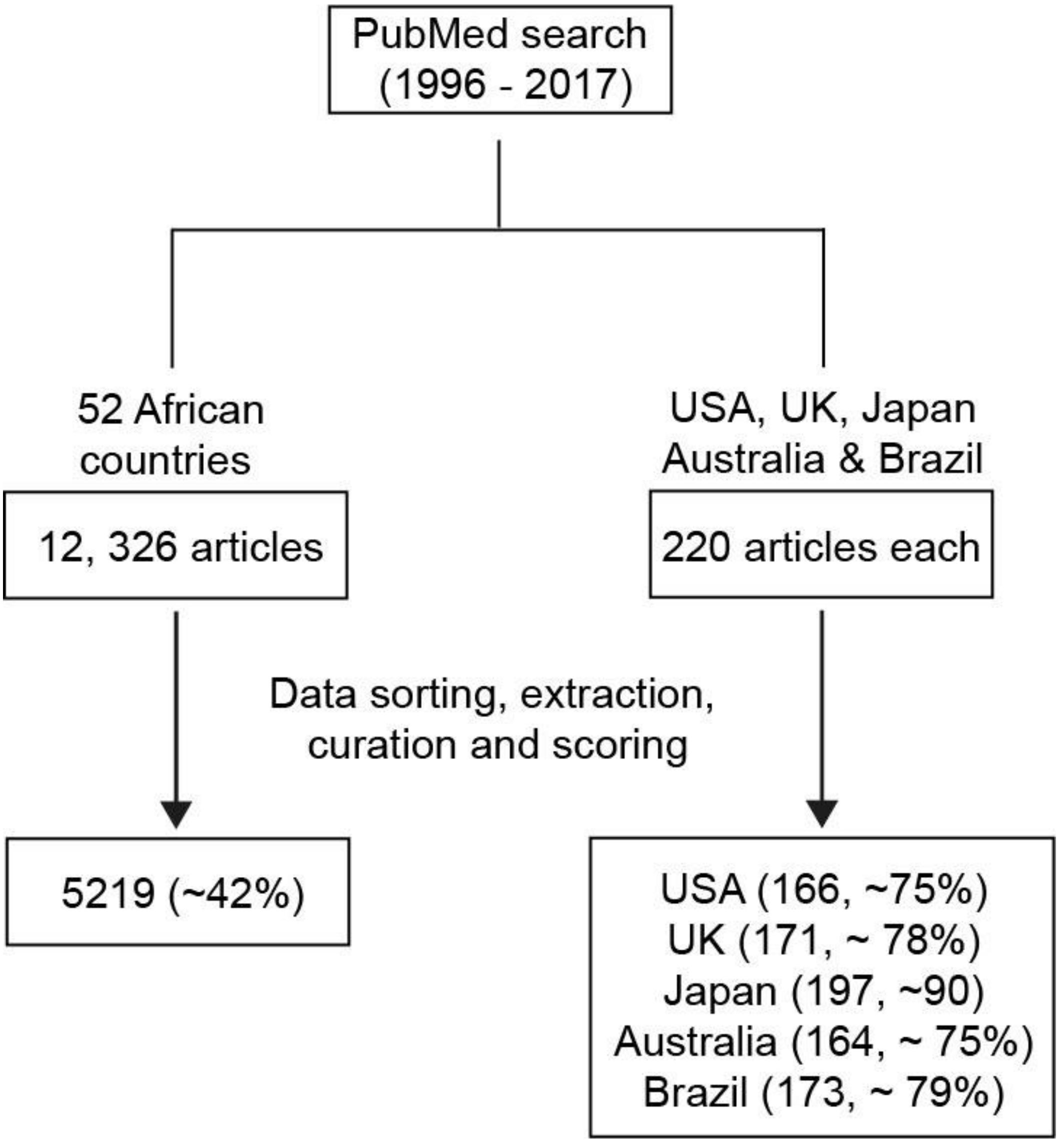

Supplementary Figure 1 | Data extraction, sorting and curation. PubMed-retrieved Neuroscience-related research articles from Africa from January 1996 to December 2017 after removal of review articles, duplicates and irrelevant articles yielded 12,326 candidate papers. Curation of these papers resulted in 5,219 papers that were used for analysis. For comparison, 220 papers each (10 papers per year) from the abovelisted non-African countries were also extracted, sorted and curated resulting in between 164-197 papers each.

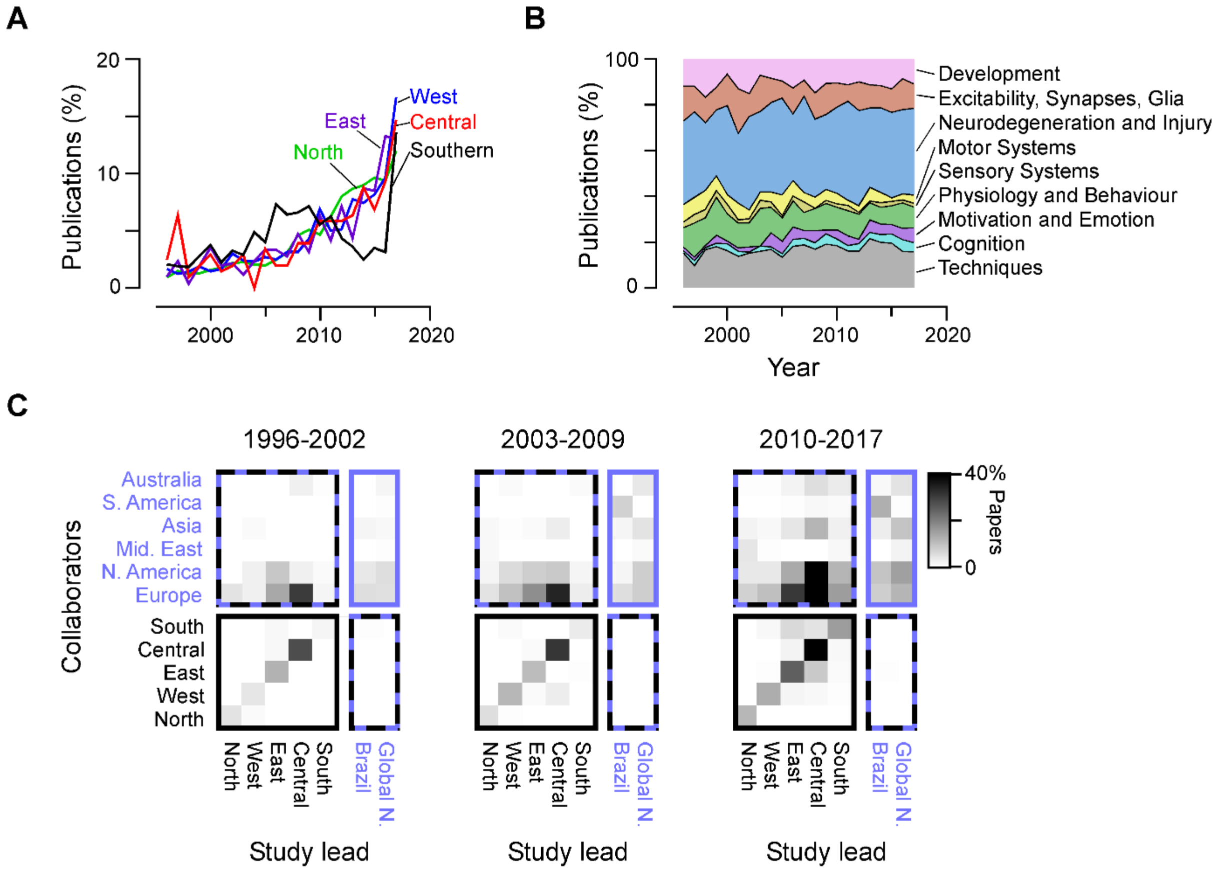

Supplementary Figure 2 | Related to Figure 1. A, Alternative representation (cf. Fig. 1B) of publication trends from Africa's five major geopolitical regions over the study period, here area-normalised per region. **B**, Alternative representation (cf. Fig. 1C) of publication trends by field of study, here plotted over time. **C**, Collaboration matrices (cf. Fig. 3B) computed for three time periods as indicated.
