# Supplementary material for: Two decades of neuroscience publication trends in Africa
Source: Nat Commun. 2021 Jun 8;12:3429. doi: 10.1038/s41467-021-23784-8 (PMC8187719; doi:10.1038/s41467-021-23784-8)
Supplement: Supplementary file 3 — Reporting summary. [file 41467_2021_23784_MOESM3_ESM.pdf]

Corresponding author(s): Tom Baden

Last updated by author(s): 2021/04/28

Reporting Summary

Nature Research wishes to improve the reproducibility of the work that we publish. This form provides structure for consistency and transparency in reporting. For further information on Nature Research policies, see our Editorial Policies and the Editorial Policy Checklist.

Statistics

For all statistical analyses, confirm that the following items are present in the figure legend, table legend, main text, or Methods section.

n/a	Confirmed	
☐	☒	The exact sample size (*n*) for each experimental group/condition, given as a discrete number and unit of measurement
☒	☐	A statement on whether measurements were taken from distinct samples or whether the same sample was measured repeatedly
☐	☒	The statistical test(s) used AND whether they are one- or two-sided<br>*Only common tests should be described solely by name; describe more complex techniques in the Methods section.*
☒	☐	A description of all covariates tested
☐	☒	A description of any assumptions or corrections, such as tests of normality and adjustment for multiple comparisons
☐	☒	A full description of the statistical parameters including central tendency (e.g. means) or other basic estimates (e.g. regression coefficient) AND variation (e.g. standard deviation) or associated estimates of uncertainty (e.g. confidence intervals)
☐	☒	For null hypothesis testing, the test statistic (e.g. F, t, r) with confidence intervals, effect sizes, degrees of freedom and P value noted<br>*Give P values as exact values whenever suitable.*
☒	☐	For Bayesian analysis, information on the choice of priors and Markov chain Monte Carlo settings
☒	☐	For hierarchical and complex designs, identification of the appropriate level for tests and full reporting of outcomes
☐	☒	Estimates of effect sizes (e.g. Cohen's *d*, Pearson's *r*), indicating how they were calculated

Our web collection on statistics for biologists contains articles on many of the points above.

Software and code

Policy information about availability of computer code

Data collection	All articles were retrieved from PubMed and curated in MS Excel 2019
Data analysis	Igor Pro version 6 (Wavemetrics), Python version 3.8

For manuscripts utilizing custom algorithms or software that are central to the research but not yet described in published literature, software must be made available to editors and reviewers. We strongly encourage code deposition in a community repository (e.g. GitHub). See the Nature Research guidelines for submitting code & software for further information.

Data

Policy information about availability of data

All manuscripts must include a data availability statement. This statement should provide the following information, where applicable:

- Accession codes, unique identifiers, or web links for publicly available datasets
- A list of figures that have associated raw data
- A description of any restrictions on data availability

Data were retrieved from PubMed at https://pubmed.ncbi.nlm.nih.gov/. All data is freely available without restriction at https://github.com/BadenLab/AfricanNeuroscience.

Field-specific reporting

Please select the one below that is the best fit for your research. If you are not sure, read the appropriate sections before making your selection.

☒ Life sciences ☐ Behavioural & social sciences ☐ Ecological, evolutionary & environmental sciences

For a reference copy of the document with all sections, see nature.com/documents/nr-reporting-summary-flat.pdf

Life sciences study design

All studies must disclose on these points even when the disclosure is negative.

Sample size

All the neuroscience-related research articles affiliated with African institutions from January 1996 to December 2017 were retrieved from PubMed and used in this study. Randomly selected 220 articles from USA, UK, Australia, Japan or Brazil (10 per year) were similarly retrieved. Compared to the number of papers retrieved for publications affiliated with African neuroscience, the 220 articles retrieved for countries outside Africa will result in a margin of error of ±7.5%, which is statistically acceptable.

Data exclusions

The search terms used to retrieve neuroscience-related research articles were "Neuroscience" OR "Nervous system" OR "Brain" OR "Neuron" OR "Spinal cord", in combination with the name of each of the individual African and non-African countries. Of these, primary research, case reports or clinical trials were included, while review articles were excluded. Next, duplicates and irrelevant articles were manually removed. This yielded a total of 12,326 candidate papers from Africa. For comparison, 220 papers each from the abovelisted non-African countries were also analysed, after randomly selecting 10 publications per year and country using the same search terms. Of these total of 1,100, n = 229 (21%) were eliminated based on the same exclusion criteria applied to our African dataset to leave a total of n = 871 non-African papers (Australia: 164; Brazil: 173; Japan: 197; UK: 171; USA: 166).

For exclusion, papers from outside of Africa were identified based on the listed affiliations of lead/corresponding/senior author(s) as well as study location. The latter was extracted from information in the materials and methods or acknowledgements, where possible. For example, articles with external collaborations in which only a small fraction of the work was conducted within Africa, such as sample collection, were excluded. This process eliminated n = 7,107 papers, leaving n = 5,219 African papers for further analysis (Fig S1).

Replication

Following the same search strategy and inclusion/exclusion strategy detailed in the manuscript, this work can be replicated.

Randomization

A random number generator was used for the selection of 10 articles per year for USA, UK, Australia, Japan or Brazil from January 1996 to December 2017 were retrieved from PubMed.

Blinding

To successfully include or exclude articles from each country, all the information related to the retrieved articles must be known during curation by the team. As a result, blinding was not possible and applicable for this study.

Reporting for specific materials, systems and methods

We require information from authors about some types of materials, experimental systems and methods used in many studies. Here, indicate whether each material, system or method listed is relevant to your study. If you are not sure if a list item applies to your research, read the appropriate section before selecting a response.

Materials & experimental systems

n/a	Involved in the study	
☒	☐	Antibodies
☒	☐	Eukaryotic cell lines
☒	☐	Palaeontology and archaeology
☒	☐	Animals and other organisms
☒	☐	Human research participants
☒	☐	Clinical data
☒	☐	Dual use research of concern

Methods

n/a	Involved in the study	
☒	☐	ChIP-seq
☒	☐	Flow cytometry
☒	☐	MRI-based neuroimaging
